# Mechanochemical Dual-Metal Modification of CuBTC Metal–Organic Frameworks for Enhanced Hydrogen Storage

**DOI:** 10.1021/acsami.5c18519

**Published:** 2025-12-08

**Authors:** Qian Yu, Charles D. Brewster, Rajan Jagpal, Arthur Graf, Xiayi Hu, Timothy J Mays, Mi Tian

**Affiliations:** † Department of Engineering, Faculty of Environment, Science and Economy, 3286 University of Exeter, Streatham Campus, Exeter EX4 4QJ, U.K.; ‡ Department of Chemical Engineering, Faculty of Engineering & Design, University of Bath, Claverton Down, Bath BA2 7AY, U.K.; § 7315 Harwellxps, Research Complex at Harwell, Harwell Campus, Didcot OX11 0FA, U.K.; ∥ Department of Chemistry, Cardiff University, Cardiff CF10 3AT, U.K.; ⊥ Chemical Engineering and Technology, Xiangtan University, Xiangtan, Hunan 411105, China

**Keywords:** metal−organic frameworks (MOFs), mechanochemical synthesis, modification of MOFs, hydrogen storage, ball milling

## Abstract

Metal–organic frameworks (MOFs) are promising materials for hydrogen storage due to their high specific surface area and structural tunability. In this study, we provide the first demonstration of enhanced hydrogen storage performance in a CuBTC (also known as HKUST-1) MOF by incorporating nickel and magnesium through a combination of *in situ* and postmodification solvent-free mechanochemical ball milling. A comprehensive combination of structural and adsorption–desorption characterization is employed to examine and understand the impact of Ni^2+^ and Mg^2+^ divalent metal ions through *in situ* and postmodification methods. In general, the post-Ni-modification method achieved higher hydrogen storage capacities than *in situ*-Ni-modification routes. The post-Ni-modified CuBTC with 30 min milling time exhibited the highest hydrogen storage capacity of 4.2 wt % at 20 bar and 77 K, which is 31% higher than the pristine CuBTC. The substitution of Cu^2+^ by Ni^2+^ during the postmodification process increased the active metal sites and Cu^+^ content, thus contributing to enhanced hydrogen storage capacity. Our findings indicate that modification via a solvent-free mechanochemical route is an effective novel strategy for improving the hydrogen storage performance of MOF materials.

## Graphical abstract

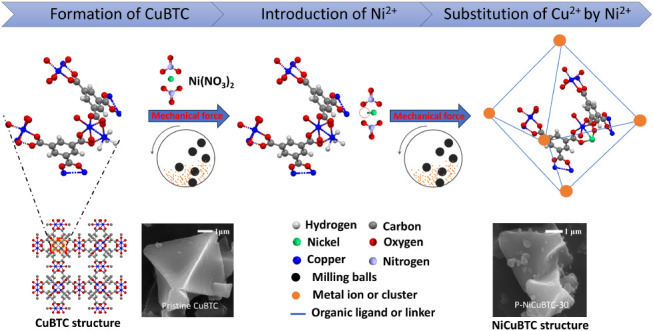

## 1 Introduction

Hydrogen (H_2_) provides considerable environmental advantages by producing zero carbon dioxide (CO_2_) emissions during utilization, supporting the transition to more sustainable and cleaner energy systems. Notably, its energy content per unit mass is approximately three times greater than that of gasoline.^1^ Therefore, advancing hydrogen technologies is essential for the development of sustainable energy systems and efficient energy carriers. To fully exploit the potential of hydrogen energy, extensive research is required in the areas of hydrogen production, distribution, storage, and fuel cell development, with hydrogen storage identified as a critical enabling technology due to its pivotal role in ensuring safe, efficient, and flexible use of hydrogen across various applications.^1^ Among the range of storage approaches, material-based hydrogen storage has attracted significant attention due to its potential to provide high-density and reversible hydrogen storage solutions suitable for both mobile and stationary energy systems.

Hydrogen storage materials are classified based on the interaction strength between the material and hydrogen, typically categorized into chemical compounds and physisorption-based systems. Chemisorption-based materials generally possess high hydrogen storage capacities;^2–4^ this category includes metal hydrides (e.g., MgH_2_, LaNi_5_H_6_) and complex hydrides such as boron hydrides (e.g., LiBH_4_) and alanates.^5^ While these materials offer significant advantages in terms of storage density and long-term storage, they are often limited by high desorption temperatures, slow kinetics, and poor reversibility.^5,6^ In particular, complex hydrides such as LiBH_4_ require temperatures above 300 °C for hydrogen release and tend to form stable byproducts that hinder rehydrogenation. In addition, many chemisorption-based systems involve high material costs and challenging synthesis or handling procedures, which currently restrict their practical applications in mobile energy systems.^7,8^

In contrast, physisorption-based materials are attractive for hydrogen storage due to their capability to operate at relatively low pressures (typically <200 bar), good cyclability, and the affordability of the constituent materials.^9^ In addition, these materials enable fully reversible hydrogen adsorption with rapid kinetics.^10^ A diverse range of porous materials has been developed for physical hydrogen storage, including metal–organic frameworks (MOFs),^11,12^ carbon-based materials,^13,14^ and polymer-based composites.^15^ MOFs, in particular, have gained significant attention due to their highly crystalline structures, tunable porous structure and high specific surface area.^16,17^ However, as the physisorption is driven by van der Waals interactions, the hydrogen storage capacity is typically dependent on cryogenic temperatures and elevated pressures.^1,18,19^

Despite this limitation, MOFs offer significant potential for optimization due to a near-limitless combination of organic linkers and metal nodes. This composition provides a high specific surface area (SSA) and chemical tunability, including adjustable pore size, modifiable framework topology, and the ability to introduce functional groups.^20,21^ These versatile features enable targeted modification strategies to improve hydrogen storage performance.^22^

The modification of MOFs to enhance hydrogen storage performance can typically be achieved by altering either the organic linkers or the metal nodes. Linker modification primarily focuses on tailoring the porous structure, including pore dimensions, geometry, and internal chemical environment.^23^ Xia et al.^24^ utilized Grand Canonical Monte Carlo (GCMC) simulations to substitute the hydrogen atom on the phenyl ring of MOF-808 with functional groups such as hydroxyl (−OH), nitrite anion (−NO_2_), methyl (−CH_3_), cyanide (−CN), and iodine (−I). These modifications demonstrated promising improvements in hydrogen storage capacity, with MOF-808-CN achieving a capacity of 3.15 wt %, representing an 11.7% increase compared to the original material due to relatively increased isosteric heat of adsorption. Similarly, Han et al.^25^ explored the introduction of hydroxyl, methyl, and ethyl groups in place of the hydrogen atom on the phenyl ring of UiO-66. Their findings revealed that UiO-66-(OCH_2_CH_3_)_2_ exhibited an improved hydrogen adsorption uptake of 4.56 wt % at 77 K and 40 bar, due to the potential energy overlap within its small cavities. However, linker functionalization typically requires multistep organic synthesis, making it time-consuming and costly.

To overcome these challenges and further enhance host–guest interactions, metal node modification offers a promising and more straightforward alternative. This approach can introduce a higher density of active sites that enhance hydrogen interactions. Huynh et al.^26^ employed GCMC simulations and demonstrated that MIL-88A substituted by scandium (Sc) achieved the highest excess hydrogen storage, reaching 4.63 wt % at 77 K and 10 bar, due to its higher isosteric heat of adsorption compared to other metals such as Sc, nickel (Ni), chromium (Cr), titanium (Ti), manganese (Mn), and vanadium­(V). Similarly, Peedikakkal and Aljundi^27^ replaced Zn^2+^ in MOF-5 with metal ions (Ni^2+^, Co^2+^, and Fe^2+^), and reported improved hydrogen storage capacities for Ni-MOF-5 and Co-MOF-5. The capacity increased to 1.53 wt % at 77 K and 1.1 bar, attributed to the presence of unsaturated accessible sites, exceeding the original MOF-5 capacity of 1.46 wt %. Despite much promise, studies focused on metal node modifications of MOFs to enhance hydrogen storage capacity are still in their infancy and remain limited. In addition, these modifications are predominantly conducted through solvent-based techniques, which generate significant solvent waste and pose environmental concerns.^28^

CuBTC (copper benzene-1,3,5-tricarboxylate, also known as HKUST-1) is considered a promising MOF candidate for hydrogen storage modification due to its well-defined pore structure, with dominant pore sizes of approximately 0.6 and 0.9 nm. Pores of this diameter range provide the highest H_2_ uptake per SSA at elevated pressures and 77 K.^29^ Specifically, the 0.6 nm pores allow two H_2_ molecules to interact simultaneously with multiple regions of the CuBTC, rather than with a single secondary building unit (SBU) or organic linker, thereby promoting the sorption energy between H_2_ and the framework.^30^ Furthermore, Letwaba et al. reported^31^ that the cage-like structure of CuBTC, with its spherical pore morphology, exposes a greater number of surface atoms, contributing to its favorable hydrogen sorption properties. This structural compatibility enables efficient confinement and adsorption of hydrogen within its pore network. Moreover, the open metal sites in CuBTC provide additional binding interactions that can enhance hydrogen uptake. These features make CuBTC an attractive platform for targeted modifications to further optimize hydrogen storage performance.^32^

In a prior study,^33^ we demonstrated the solid-state synthesis of CuBTC via a solvent-free mechanochemical approach, achieving comparable quality to conventional solvent-based methods but achieved in only minutes, with high yield, and minimal solvent use. However, the physisorptive hydrogen storage properties need to be improved to make the material suitable for practical application. The present work therefore pioneers a novel approach to metal modification of CuBTC through mechanochemical ball milling. Using detailed characterization and by incorporating divalent metal ions (Mg^2+^ and Ni^2+^) into the CuBTC framework and by employing both *in situ* and postsynthetic ball milling approaches, we demonstrate an innovative and efficient pathway to enhance the hydrogen storage properties of MOFs. Among the metal modifications investigated in this study, Ni^2+^ introduction resulted in the most significant enhancement in hydrogen storage performance and isosteric enthalpy compared to pristine CuBTC. This improvement is attributed to the increase of the additional active metal adsorption sites and concentration of Cu^+^ species, resulting from the substitution of Cu^2+^ by Ni^2+^.

To the authors’ knowledge, this study represents the first report of Ni^2+^ doping via mechanochemical ball milling, and demonstrates a novel strategy to tune MOF composition, resulting in improved hydrogen uptake. As a result, our work highlights the promise of dual-metal frameworks, such as NiCuBTC, in overcoming the current limitations in term of physisorptive hydrogen storage and brings us closer to practical, scalable solid-state hydrogen storage solutions. This also demonstrates that targeted mechanochemical metal modification can be used to tune the electronic environment of open metal sites in CuBTC, providing a practical route for improving hydrogen storage performance.

## 2 Experimental Methods

### 2.1 Synthesis Procedures

#### 2.1.1 Materials

Copper­(II) nitrate hemi­(pentahydrate) (Cu­(NO_3_)_2_·2.5H_2_O), benzene-1,3,5-tricarboxylic acid (HBTC), and dimethyl sulfoxide (DMSO), magnesium nitrate hexahydrate (Mg­(NO_3_)_2_·6H_2_O), nickel nitrate hexahydrate (Ni­(NO_3_)_2_·6H_2_O) were purchased from Merck without further purification. The zero-grade nitrogen (N_2_), CP grade 99.999% hydrogen (H_2_), and CP grade 99.999% helium (He) were purchased from BOC Limited.

All milling was conducted using a Frisch micro mill pulverizette 7, using stainless steel milling jars and balls.

#### 2.1.2 Pristine CuBTC Synthesis

The pristine CuBTC was synthesized following the method established in our previous study,^33^ serving as the benchmark material throughout this study.

Pristine CuBTC was synthesized using a mechanochemical ball milling approach with a mass ball to powder ratio of 7.5 w/w. Specifically, an 80 mL stainless steel milling jar was loaded with 1 ball of 15 mm diameter, 5 balls of 10 mm diameter, and 41 balls of 5 mm diameter, all made of hardened stainless steel. Subsequently, 4.904 g of Cu­(NO_3_)_2_·2.5H_2_O (0.0211 mol), 2.332 g of HBTC (0.0111 mol), and 0.1 mL of DMSO were added to the milling jar. The jar was then mounted on the ball milling machine of the planetary micro mill pulverizette 7 machine and milled at 280 rpm for 10 min, followed by 30 min rest before the next steps. Subsequently, the resulting product was washed with methanol and dried at room temperature. Then the resulting material is referred to as “*pristine CuBTC*” and is used for further modification and as a reference sample for comparison.

#### 2.1.3 *In Situ* Metal Node Modification

The *in situ* modification of CuBTC was performed using a mechanochemical ball milling approach with a mass ball to powder ratio of 7.5 w/w. An 80 mL stainless steel milling jar was loaded with 2 balls of 15 mm diameter, 6 balls of 10 mm diameter, and 47 balls of 5 mm diameter, all made of hardened stainless steel. Subsequently, 2.332 g of HBTC (0.0111 mol), 4.904 g of Cu­(NO_3_)_2_·2.5H_2_O (0.0211 mol), 2.703 g of Mg­(NO_3_)_2_·6H_2_O (0.0105 mol), and 0.1 mL of DMSO were added to the milling jar. The jar was then mounted on the ball milling machine and milled at 280 rpm for 10 min to obtain the magnesium modified CuBTC sample (I-MgCuBTC).

For the synthesis of the *in situ* nickel modified CuBTC sample (I-NiCuBTC) with a mass ball to powder ratio of 7.5 w/w, a similar procedure was followed, with slight variations: the milling media consisted of 2 balls of 15 mm, 7 balls of 10 mm, and 44 balls of 5 mm in diameter. In this case, 3.066 g of Ni­(NO_3_)_2_·6H_2_O (0.0105 mol) was used in place of the magnesium nitrate.

#### 2.1.4 Post Synthesis Modification Procedure

In the post modification process, pristine CuBTC was synthesized and used directly without being heated, serving as the base material for subsequent modification.

For the post magnesium modification, 1 milling ball of 10 mm in diameter, and 4 balls of 5 mm were loaded into the 80 mL stainless steel milling jar for the ball to powder ratio of 8. Then 0.5 g of pristine CuBTC (before activation) and 0.25 g Mg­(NO_3_)_2_·6H_2_O (9.75 × 10^–3^ mol) were introduced to this milling jar. The jar was then placed on the milling machine and milled at 280 rpm for 10 min to produce the P-MgCuBTC sample.

For the preparation of postmodified P-NiCuBTC samples, the same milling conditions were applied with some variations: 0.25 g of Ni­(NO_3_)_2_·6H_2_O (8.60 × 10^–3^ mol) was used instead of Mg­(NO_3_)_2_·6H_2_O. To study the effect of milling duration, samples were prepared with milling times of 10, 20, 30, and 40 min, resulting in the P-NiCuBTC-10, P-NiCuBTC-20, P-NiCuBTC-30, and P-NiCuBTC-40 samples, respectively.

For all modified samples in this work, after the finish of the milling process, the samples were left to rest in the milling jar for 30 min before opening. The solid products were then washed by centrifugation at 8000 rpm using methanol, air-dried at room temperature, and activated in an oven at 170 °C for 7 h.

### 2.2 Characterization Methods

Powder X-ray diffraction (XRD) was used to analyze the crystal structure of the synthesized powder samples using a Bruker D8 Advance diffractometer. The instrument was operated at 40 kV and 40 mA using Cu–Kα radiation (λ=1.5406 Å). The samples were pressed into a flat form and measured at room temperature. Data were collected over a 2θ range of 5° to 25°, using a step size of 0.04° at a rate of 1 step per second.

To gain insight into the functional groups present within the samples, Fourier-transform infrared spectroscopy (FTIR) was recorded using a Shimadzu IRTracer-100 spectrometer over the wavelength range of 2000 to 500 cm^– 1^. The measurements were performed on samples prepared as pressed disks mixed with KBr.

Scanning electron microscopy (SEM) equipment with a focused ion beam (FIB) was used to examine the morphology of the samples, which were obtained using a Helios Nanolab 600i DualBeam system. SEM equipment with an X-MAXN energy-dispersive X-ray spectroscopy (EDS) detector was applied for elemental composition^34^ using a TESCAN VEGA3. Dried powder samples were lightly sprinkled onto carbon tape and stuck on a sample holder. To enhance conductivity, the samples were coated with an 8 nm layer of chromium (Cr) before imaging.

Transmission Electron Microscopy (TEM) is a technique in which a beam of electrons passes through the samples to generate high-resolution images. TEM and EDS analysis in this study was performed using a JEOL-2100 LaB_6_ transmission electron microscope to clarify the elemental distribution. Prior to imaging, the samples were dispersed in ethanol and ultrasonicated to ensure uniform suspension. A drop of the suspension was then placed onto a copper grid and allowed to dry at room temperature. The dried grid containing the sample was subsequently used for TEM and EDS measurement.

Thermogravimetric analysis (TGA) combined with derivative thermogravimetry (DTG) was carried out using a TA 55 instrument to obtain information about the thermal stability of the sample. The furnace was calibrated using a nickel standard to ensure temperature accuracy. The measurements were conducted from 50 to 900 °C at a heating rate of 10 °C min^–1^ under a N_2_ and air flow of 60 mL min^–1^. Before the test, the samples were treated in an isothermal step at a nitrogen flow rate of 200 mL min^–1^ for 1 h to minimize the risk of oxidation under the N_2_ atmosphere.

N_2_ adsorption–desorption measurements were performed at 77 K using a Quantachrome AutosorbiQ gas sorption analyzer. Prior to the analysis, approximately 40 mg of the sample was degassed *in situ* at 150 °C for 15 h. The Brunauer–Emmett–Teller (BET) specific surface area was calculated in the relative pressure (P/P_0_) range of 0.01 to 0.35, following the Rouquerol criteria.^35^ The total pore volume was estimated at a relative pressure of approximately 0.95. Pore size distribution was analyzed using Non-Local Density Functional Theory (NLDFT) based on the full adsorption isotherm.

Gravimetric hydrogen sorption isotherms were measured at 77 K and up to 20 bar using an Intelligent Gravimetric Analyzer-003 (IGA, Hiden Isochema). Before the measurement, ∼50 mg samples were loaded and then outgassed under dynamic vacuum (<1 × 10^–6^ mbar) at 120 °C for 12 h to remove residual impurities, including moisture and other adsorbed species. Buoyancy correction was applied following Archimedes’ principle using the skeletal volume determined via a method analogous to helium pycnometry.

High-pressure volumetric sorption isotherms were collected on an HTP-1 (Hiden) sorption analyzer at 77 K, 87 K, 97 K and up to 150 bar. Prior to hydrogen uptake measurements, approximately 150 mg of each sample was degassed *in situ* at 120 °C under vacuum (<1 × 10^–6^ mbar) overnight to remove moisture and other adsorbed species. To remove potential residual adsorbed hydrogen between temperature changes, an additional 2 h degassing step was conducted between isotherms.

The isosteric enthalpy of adsorption was determined using the Clausius–Clapeyron equation based on the isotherms collected at the three temperatures. The absolute hydrogen uptake was first calculated from parameters obtained via fitting of the measured excess isotherms at each temperature using the Sips model. For a given value of absolute uptake, the isosteric enthalpy was then obtained from the gradient of the ln­(P) versus 1/T isosteres.^36,37^

Composition and speciation were determined by X-ray photoelectron spectroscopy (XPS)^38^ and were performed at the EPSRC National Facility at the Rutherford Appleton Laboratory. XPS data was acquired using a Kratos Axis SUPRA using monochromated Al kα (1486.69 eV) X-rays at 15 mA emission and 12 kV HT (180 W) and a spot size/analysis area of 700 × 300 μm. The instrument was calibrated to gold metal Au 4f (83.95 eV) and dispersion adjusted give a BE of 932.6 eV for the Cu 2p3/2 line of metallic copper. Ag 3d_5/2_ line fwhm at 10 eV pass energy was 0.544 eV. Source resolution for monochromatic Al Kα X-rays is ∼0.3 eV. The instrumental resolution was determined to be 0.29 at 10 eV pass energy using the Fermi edge of the valence band for metallic silver. Resolution with charge compensation system on <1.33 eV fwhm on PTFE. High resolution spectra were obtained using a pass energy of 20 eV, step size of 0.1 eV and sweep time of 60 s, resulting in a line width of 0.696 eV for Au 4f_7/2_. Survey spectra were obtained using a pass energy of 160 eV. Charge neutralization was achieved using an electron flood gun with filament current = 0.4 A, charge balance = 2 V, filament bias = 4.2 V. A survey scan (0–1400 eV) of the sample surface was conducted, followed by high-resolution scans in the binding energy ranges of 871–850 eV for Ni, 950–930 eV for Cu, 536–528 eV for O, and 292–282 eV for C. All spectra were charge-shifted to the C 1S peak at 284.8 eV before analysis. Data was analyzed using CasaXPS v2.3.19PR1.0. Peaks were fit with a Shirley background prior to component analysis. Lineshapes of LA (1.53,243) were used to fit components. FHWM of all peaks were the same for all elements in the same orbital, unless exceptions apply (i.e., metal oxide peaks).

## 3 Structural Characterization of Modification Samples

In our previous work,^33^ the effects of ball milling parameters (milling time, speed, ball-to-powder ratio, starting material weight, and DMSO amount) on the synthesis of CuBTC were systematically evaluated. The results showed that the ball milling time alone did not lead to significant changes in the structural or adsorption properties of CuBTC. Therefore, the structural and adsorption differences observed in Ni-CuBTC and Mg-CuBTC are attributed to the presence of Ni^2+^ and Mg^2+^ during the mechanochemical ball milling modification process.

The XRD patterns of all metal-modified samples are presented in Figure 1, confirming the structure of both *in situ* and postmodified CuBTC (HKUST-1) typically crystallizes in a face-centered cubic (FCC) structure with Cu paddle-wheel clusters connected by benzene-1,3,5-tricarboxylate linkers, forming a 3-dimensional porous framework with two types of cages—large (∼0.9 nm) and small (∼0.6 nm)—that are accessible via square windows. Most of the metal modified samples retain the characteristic diffraction peaks at 2θ of 9.5°, 11.6°, 13.4°, 14.6°, 15°, 16.4°, 17.4°, and 19°, 20.2°, 21.3°, corresponding to the (220), (222), (400), (331), (420), (422), (511), (440), (600), and (620) planes, respectively. The XRD intensities are enhanced after Mg^2+^ and Ni^2+^ modification. However, the noticeable peaks at 2θ = 5.8° (111) and 6.7° (200) suggest the partial loss of the FCC crystal structure of the sample as a result of hydration.^39,40^

**Figure 1:**
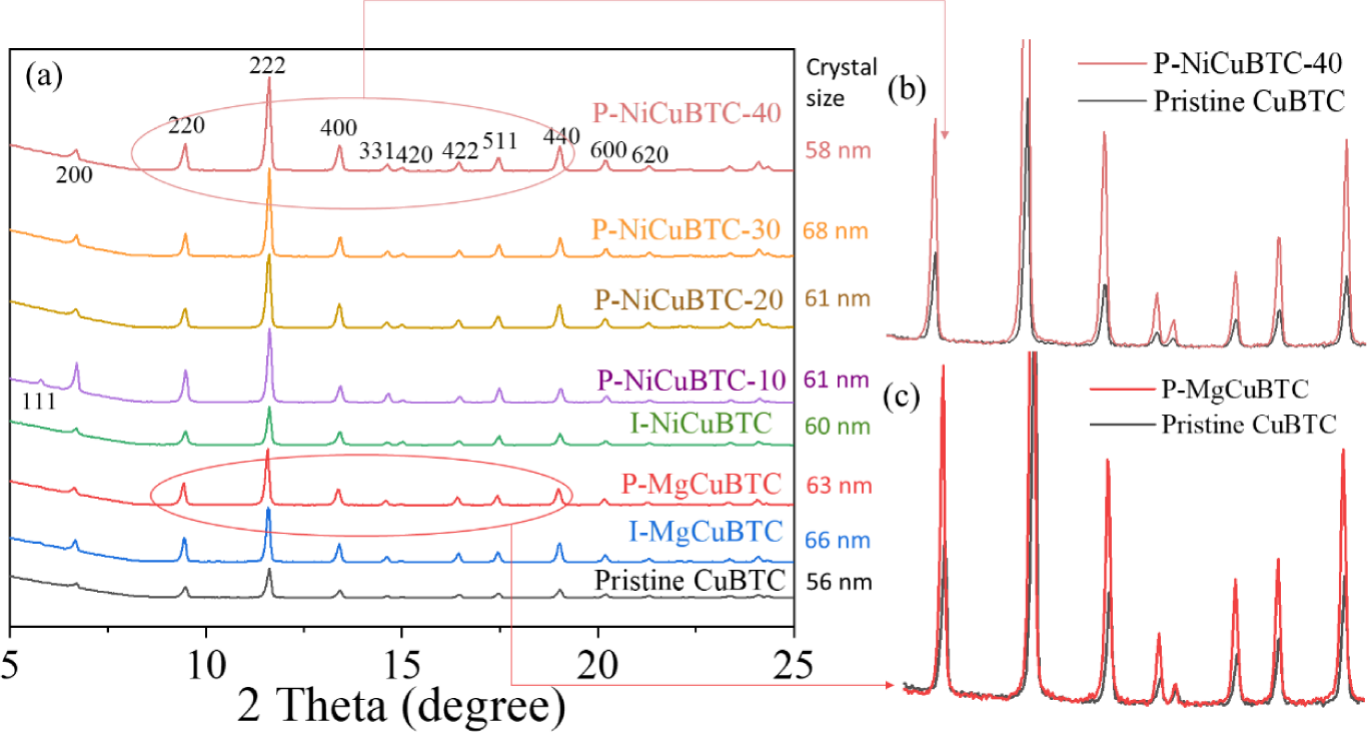
XRD diffractograms of (a) all samples, XRD comparison of (b) postnickel modified CuBTC (P-NiCuBTC-40) and (c) postmagnesium modified CuBTC (P-MgCuBTC) with pristine CuBTC.

Notably, the P-NiCuBTC-40 and P-MgCuBTC samples displayed broader diffraction peaks than pristine CuBTC, along with a slight shift to lower 2 Theta in their seven main diffraction peaks, as seen in Figure 1b,c. These diffraction peak changes suggest alterations in the crystal lattice structure caused by postmodification.^41^

The crystal size of all metal-modified CuBTC samples showed differences following the modification process. Notably, the P-NiCuBTC-30 has the largest crystal size of 68 nm, which showed a 21% increase compared to the 56 nm crystal size of pristine CuBTC.

The FTIR spectra of all samples are presented in Figure 2. The pristine CuBTC exhibited five strong characteristic peaks at 729, 759, 1373, 1448, and 1647 cm^–1^. The peaks at 729 and 759 cm^–1^ are attributed to the scissoring vibrational mode between the carboxylate ion and metals, confirming the successful formation of pristine CuBTC.^42,43^ The intense bands observed at 1373, 1448, and 1647 cm^–1^ correspond to the stretching vibrations within the organic linker structure.^44,45^ Notably, following modification with Mg^2+^ and Ni^2+^ ions, the characteristic absorption features showed no discernible change, suggesting that the introduced amount of metal ions was insufficient to alter the primary coordination environment of the CuBTC framework within the current FTIR resolution.

**Figure 2:**
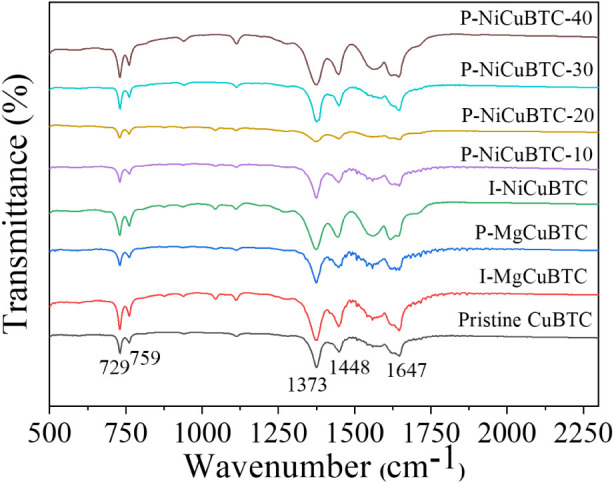
Fourier-transform infrared spectroscopy (FTIR) patterns of all samples.

TGA was performed on all samples, as shown in Figure 3 under a N_2_ atmosphere and Figure 4 under an air atmosphere. The initial weight loss observed below 150 °C is attributed to the evaporation of physically adsorbed water and other solvents,^46^ a common feature in MOF materials due to their porous nature. This low-temperature loss is followed by a more substantial weight drop, corresponding to the thermal decomposition of the organic linkers and decomposition of the CuBTC framework under N_2_ and air atmospheres.

**Figure 3:**
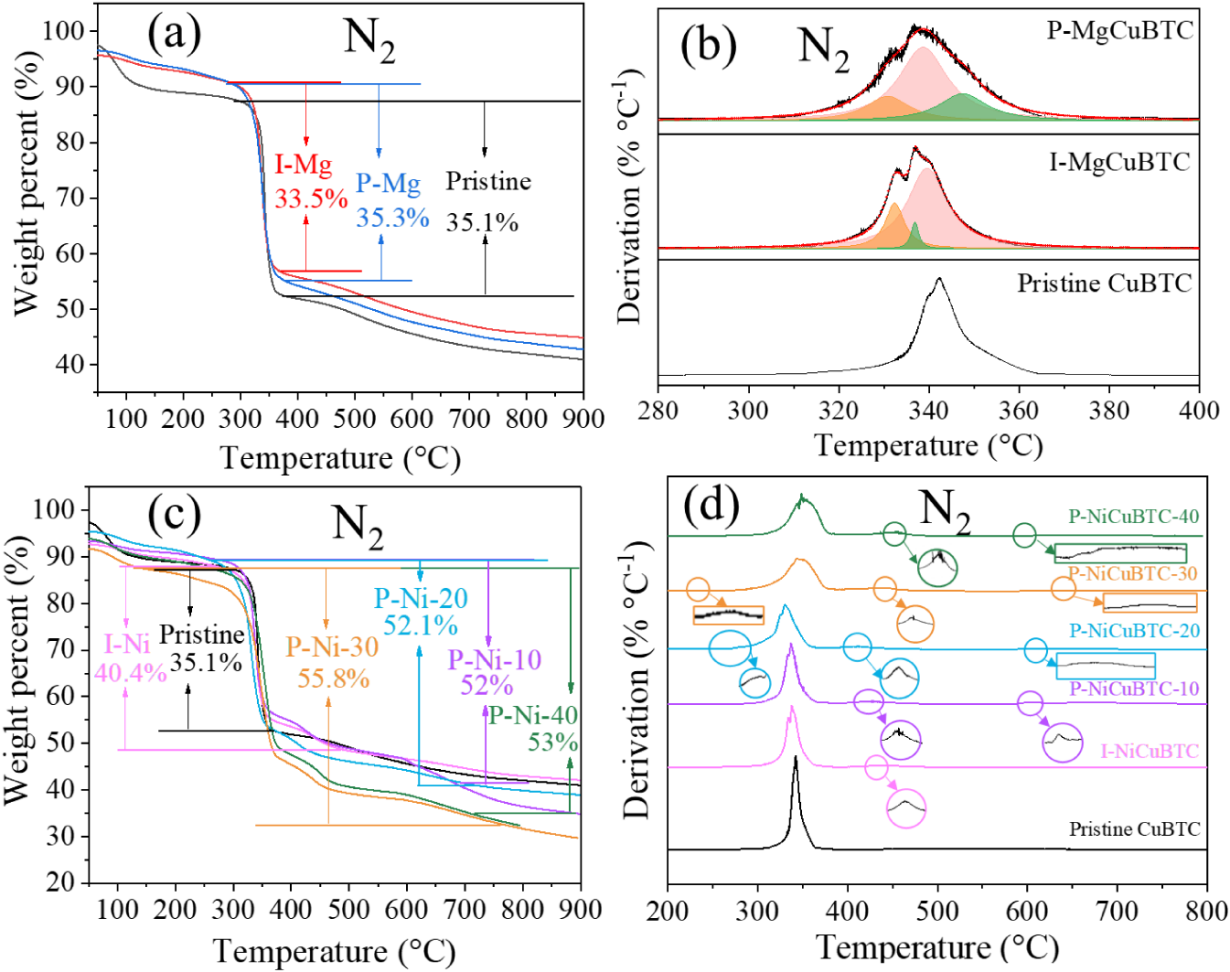
(a) TGA analysis and (b) derivative thermogravimetry (DTG) of pristine and Mg-modified CuBTC; (c) TGA analysis and (d) DTG analysis of pristine and Ni-modified CuBTC under N_2_ atmosphere.

**Figure 4:**
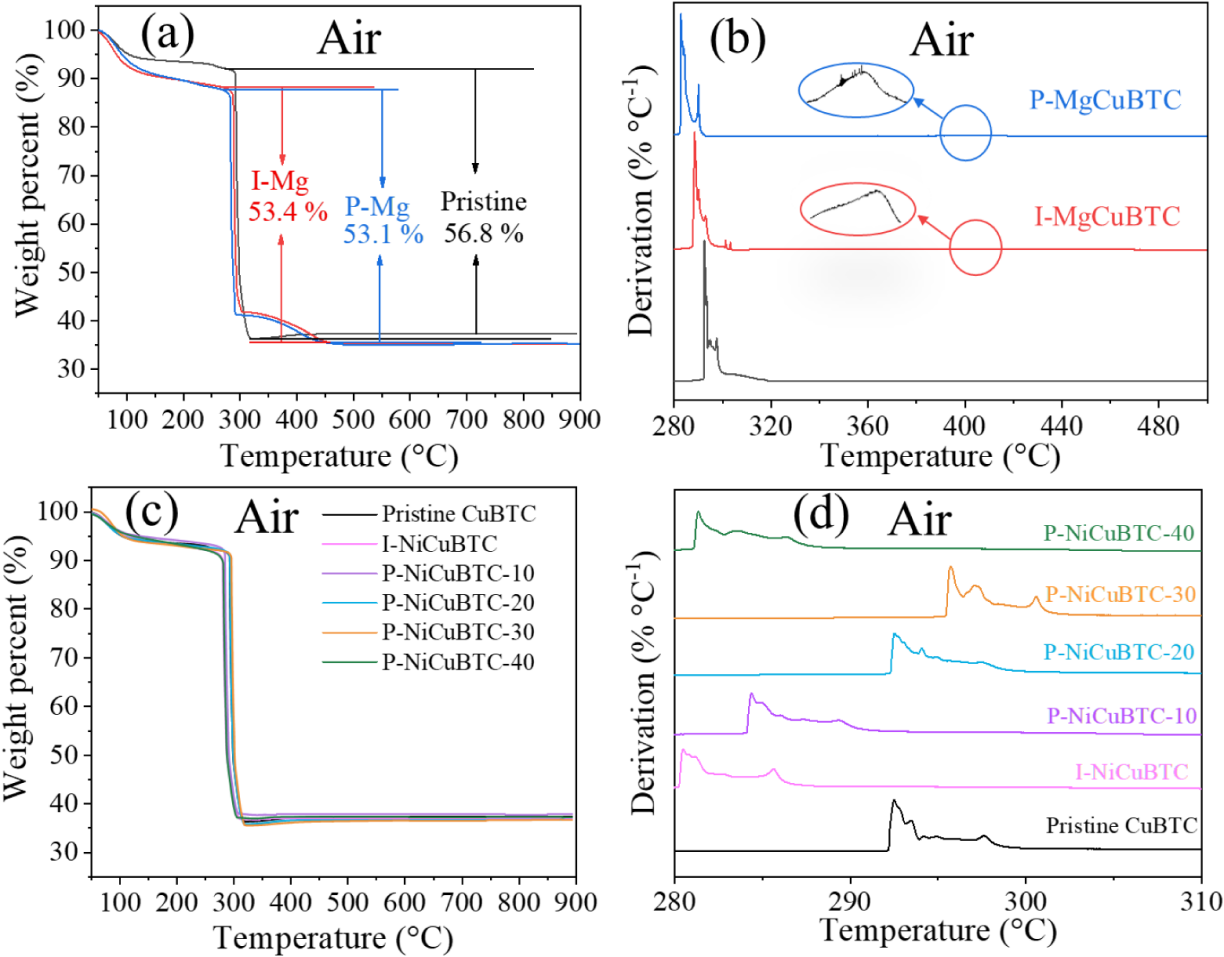
(a) TGA analysis and (b) derivative thermogravimetry (DTG) of pristine and Mg-modified CuBTC; (c) TGA analysis and (d) DTG analysis of pristine and Ni-modified CuBTC under an air atmosphere.

As shown in Figure 3a, the two Mg-modified CuBTC samples, P-MgCuBTC and I-MgCuBTC exhibit a single major weight loss of approximately 35% under N_2_ atmosphere. In addition, the DTG results in Figure 3b reveal that the decomposition peak observed in pristine CuBTC splits into three overlapping peaks in the Mg-modified samples. This suggests the introduction of Mg^2+^ alters the decomposition of CuBTC, resulting in a three-step characteristic decomposition process. Moreover, the structural decomposition occurs at a lower temperature in the Mg-modified samples compared to pristine CuBTC, indicating a slight reduction in thermal stability likely caused by Mg^2+^ introduction, while still maintaining the overall framework integrity as confirmed by XRD.

In contrast, the Ni-modified CuBTC series exhibits different thermal behavior, as shown in Figure 3c under N_2_ atmosphere. All Ni-modified CuBTC samples exhibit a greater total weight loss compared to pristine CuBTC. This increase might be attributed to the partial substitution of Cu^2+^ by Ni^2+^ within the CuBTC framework and ball milling modification process, which likely induces structural defects. The characteristic DTG peaks of Ni­(NO_3_)_2_ in N_2_ (typically observed near 200–250 °C^47^) are not present in the Ni-modified samples. Therefore, the increased weight loss cannot be attributed to residual Ni­(NO_3_)_2_, but rather confirms that Ni^2+^ successfully substituted Cu^2+^ within the CuBTC structure. With increasing postmodification time, the total weight loss of the Ni-modified CuBTC samples generally increases, but a decrease is observed for P-NiCuBTC-40, which may be attributed to partial degradation of the framework during the ball milling modification process, as evidenced by the broadening and shifting of the XRD peaks. In general, the post-Ni-modification route leads to greater weight loss compared to the *in situ* Ni-modification, indicating that post-Ni-modification generally introduces a higher degree of Cu^2+^ substitution by Ni^2+^ and generates more structural defects. Particularly, P-NiCuBTC-30 exhibits the highest degree of Cu^2+^ substitution by Ni^2+^ and defect formation among the Ni-modified samples, as indicated by its greatest weight loss.

The DTG results in Figure 3d support these observations by revealing multiple thermal events that are absent in pristine CuBTC. These additional thermal decompositions, corresponding to the increased weight loss in the Ni-modified samples, suggest the presence of new bound Ni^2+^ species that decompose at temperatures distinct from those of the bulk CuBTC framework.

In addition, Ni^2+^ introduction affects the decomposition onset temperature of the samples as illustrated in Figure 3d. For I-NiCuBTC, the decomposition at a lower temperature of 337 °C compared to pristine CuBTC of 342 °C indicates reduced thermal stability. In P-NiCuBTC series samples, decomposition gradually shifts to higher temperatures with increasing modification time. P-NiCuBTC-30 and P-NiCuBTC-40 decompose at temperatures of 346 and 350 °C, respectively, exceeding that of pristine CuBTC. This observation suggests that extended postmodification time can strengthen the framework thermally through gradual structural reorganization.^48,49^ Furthermore, the post-Ni-modification method generally provides higher thermal stability compared to the *in situ*-Ni-modification method. Among all Ni-modified samples, P-NiCuBTC-30 exhibits the highest Ni^2+^ concentration and has relatively enhanced thermal stability.

As shown in Figure 4a,b, the Mg-modified CuBTC sample exhibits an additional peak at approximately 300–450 °C in air. This temperature range corresponds to the thermal decomposition of Mg­(NO_3_)_2_ in air,^50^ indicating that Mg^2+^ may not incorporate into the CuBTC framework and that part of the Mg­(NO_3_)_2_ precursor remained after methanol washing, likely due to its low solubility in methanol.

In contrast, as shown in Figure 4c,d, the Ni-modified CuBTC samples display a typical CuBTC-type TGA profile under air during the oxidation process, with a total weight loss of approximately 55% and without any additional decomposition peaks. This further confirms that Ni^2+^ successfully substituted part of the Cu^2+^ in the framework, forming a bimetallic Cu–Ni MOF, consistent with the N_2_ TGA results. If residual Ni­(NO_3_)_2_ were present, characteristic DTG peaks would appear in air near 150–250 °C and 280–350 °C,^51^ which are not observed here. The DTG results also show that P-NiCuBTC-30 exhibits the highest thermal stability in air among all the samples, indicating that the substitution of Ni^2+^ enhances the thermal stability of the CuBTC framework.

SEM-EDS was performed on all modified samples to determine the Mg^2+^ and Ni^2+^ contents, as shown in Table 1 and Figure S1. The Mg^2+^ content was detected but was significantly lower than the Ni^2+^ content. In the Ni-modified samples, the postmodification route results in a higher Ni^2+^ content compared to *in situ* modification, which is consistent with the larger weight loss observed under N_2_ in the TGA analysis. Furthermore, during the postmodification process, the Ni^2+^ content increases slightly with the increase of the ball-milling modification time, matching the gradual increase in weight loss and thermal stability. This indicates that the increase in modification time promotes Cu^2+^ substitution by Ni^2+^ and the formation of Ni–Cu dual-metal MOFs. However, at 40 min of ball milling modification time, a decrease in Ni^2+^ content is observed, which is attributed to partial structural degradation, as evidenced by the broadened and shifted XRD peaks.

**Table 1:** Mg^2+^ and Ni^2+^ Content of All the Modified CuBTC Samples from SEM-EDS Results

Sample name	Mg^2+^or Ni^2+^concentration (wt %)	Cu^+/2+^content (wt %)	Mg^2+^or Ni^2+^ratio (%)
I-MgCuBTC	0.3	24.7	1.2
P-MgCuBTC	0.5	30.4	1.6
I-NiCuBTC	0.5	15.5	3.1
P-NiCuBTC-10	1	17.5	5.4
P-NiCuBTC-20	1.3	22.8	5.4
P-NiCuBTC-30	1.5	23.8	5.9
P-NiCuBTC-40	1.1	22	4.8

TEM and EDS analysis were conducted on I-MgCuBTC, the Mg-modified sample that did not show structural degradation, and on P-NiCuBTC-30, which contains the highest Ni^2+^ concentration of 1.5 wt.%. As shown in Figure 5, pristine CuBTC was examined as a reference. It displays the typical CuBTC particle morphology, and the corresponding EDS elemental maps of Cu, C, and O indicate a uniform composition.

**Figure 5:**
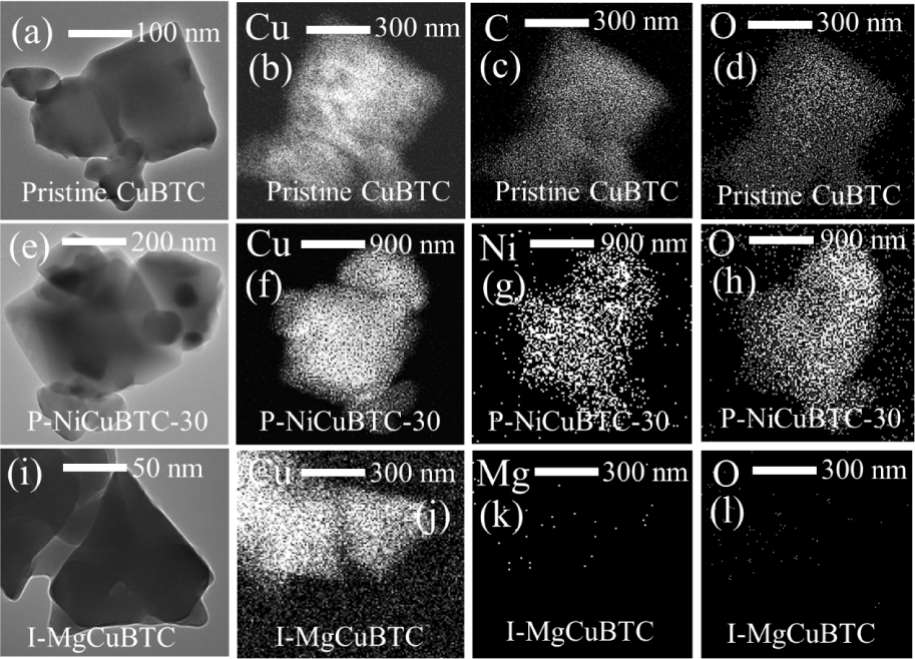
(a) TEM image of pristine CuBTC and corresponding EDS elemental maps of (b) Cu, (c) C, and (d) O; (e) TEM image of P-NiCuBTC and EDS maps of (f) Cu, (g) Ni, and (h) O; (i) TEM image of I-MgCuBTC and EDS maps of (j) Cu, (k) Mg, and (l) O.

The P-NiCuBTC-30 sample retains the characteristic CuBTC morphology, which is consistent with its unchanged XRD pattern. Notably, the TEM-EDS mapping of the P-NiCuBTC-30 particle in Figure 5g shows that Ni^2+^ is homogeneously distributed in accordance with the CuBTC framework rather than randomly. This observation supports the TGA results that Ni^2+^ is partially substituted for Cu^2+^ within the CuBTC structure. However, the Ni/Cu ratio obtained from TEM-EDS (Table S1) appears lower than expected, likely due to interference from the copper support grid. Therefore, the actual Ni/Cu ratio within the particles should be higher, as suggested by the SEM-EDS measurement of approximately 5.9% (1.5 wt % concentration).

For the I-MgCuBTC sample, most particles adhered to the copper grid edge, as shown in Figure S2. A particle located away from the grid edge (Figure 5i) displays a rougher surface compared to pristine CuBTC. In addition, no Mg^2+^ signal was detected by TEM-EDS in this sample, whereas SEM-EDS from a big picture confirmed the presence of Mg^2+^. This phenomenon supports the TGA analysis that Mg^2+^ was not incorporated into the CuBTC framework but remained as an external Mg^2+^ species.

The N_2_ adsorption and desorption isotherms at 77 K were measured on all modified samples to assess their porosity and SSA as presented in Figure 6. Figure 6a illustrates that all samples exhibit a characteristic dual-step isotherm pattern of CuBTC. The pore size distribution in Figure 6b reveals two main pore sizes at around 0.6 and 0.9 nm, which is consistent with the features observed in the isotherm curves in Figure 6a.

**Figure 6:**
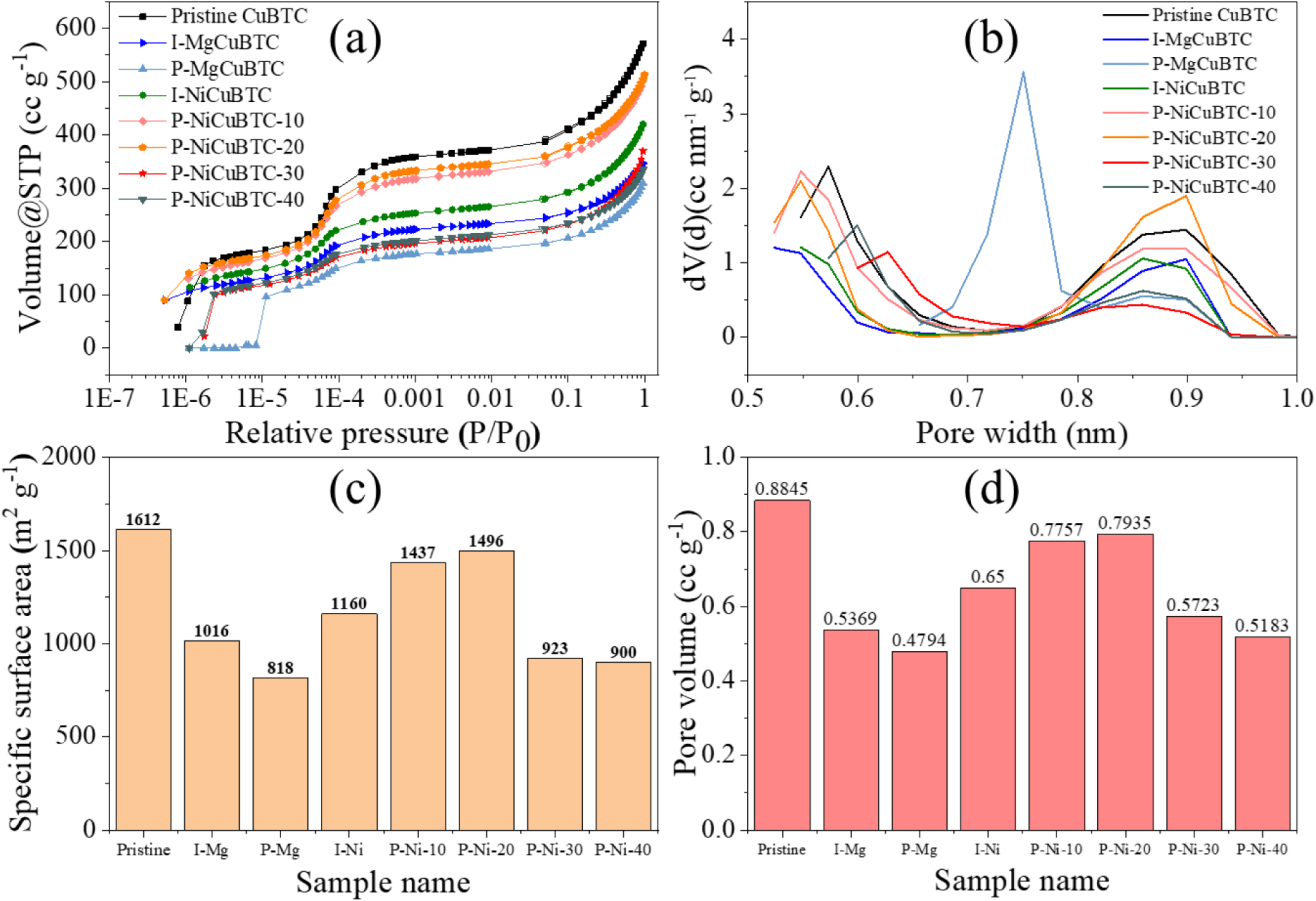
(a) Logarithmic N_2_ adsorption (filled data markers) and desorption (hollow data markers) isotherm at 77K, (b) pore size distribution, (c) BET specific surface area, and (d) pore volume of all modified samples.

In the Mg-modified samples, I-MgCuBTC exhibits a reduced N_2_ adsorption capacity, indicating a lower number of pores of similar size relative to pristine CuBTC. This is accompanied by a decreased SSA and total pore volume. These observations suggest that the introduction of Mg^2+^ during synthesis may interfere with the CuBTC framework formation, leading to a less developed porous structure. This is further supported by DTG results, which show reduced thermal stability for I-MgCuBTC compared to pristine CuBTC.

For P-MgCuBTC, the introduction of Mg^2+^ appears to have caused partial collapse or distortion of the pore structure, resulting in the lowest SSA and total pore volume among all samples. This is evidenced by a significant reduction in the pore volume around 0.9 nm (see Figure 6b), and a shift of the 0.6 nm pore size to approximately 0.75 nm. Correspondingly, the XRD pattern of P-MgCuBTC shows broader and shifted diffraction peaks, while its DTG curve reveals reduced thermal stability compared to pristine CuBTC, both of which point to increased structural disorder and degradation.

All Ni-modified samples exhibit two main pore sizes at around 0.6 and 0.9 nm. However, they show reduced SSA and total pore volumes compared to pristine CuBTC, which may be attributed to the substitution of Cu^2+^ by Ni^2+^ and structural defects, as suggested by TGA results and supported by SEN-EDS and TEM-EDS results. Among the Ni-modified samples, P-NiCuBTC-40 displays the lowest SSA and pore volume, likely due to partial framework collapse, as indicated by the broadening of XRD peaks.

Across all modified samples, the introduction of secondary metal ions results in lower SSA and total pore volume compared to the pristine CuBTC (1612 m^2^ g^–1^ and 0.8845 cc g^–1^) as seen in Figure 6c,d. In addition, textural degradation induced by the modification process during ball milling also contributes to the decline in porosity,^41,52^ a phenomenon discussed in greater detail in the subsequent SEM analysis.

SEM images of all samples are presented in Figure 7. Figure 7a shows the well-defined, smooth octahedral pristine CuBTC, which serves as the reference for evaluating the effects of metal modification.

**Figure 7:**
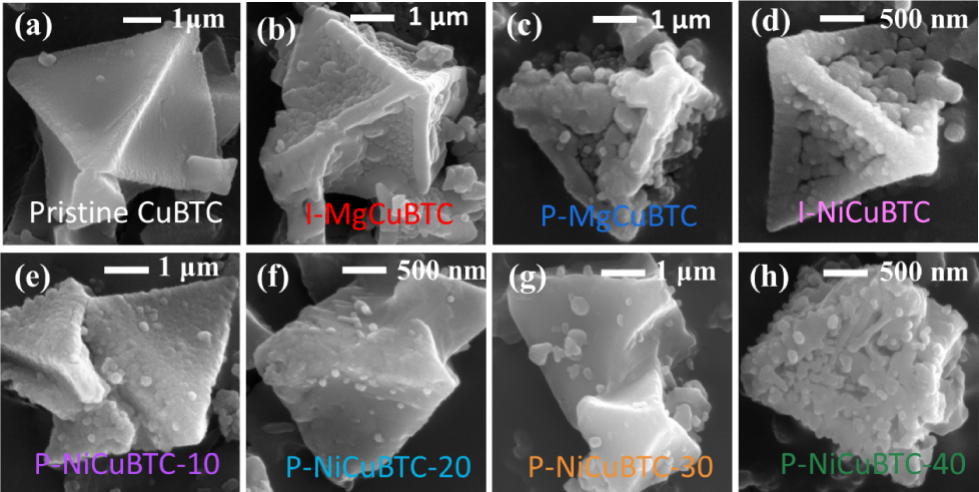
SEM images of samples (a) pristine CuBTC, (b) *in situ* magnesium modified I-MgCuBTC, (c) magnesium postmodified P-MgCuBTC, (d) *in situ* nickel modified I-NiCuBTC, (e) nickel postmodified for 10 min, P-NiCuBTC-10, (f) nickel postmodified for 20 min, P-NiCuBTC-20, (g) nickel postmodified for 30 min, P-NiCuBTC-30, and (h) nickel postmodified for 40 min, P-NiCuBTC-40.

As supported by TGA and N_2_ sorption analysis, the *in situ* introduction of Mg^2+^ interferes with the crystallization process of the CuBTC, resulting in less well-developed structures. This observation is reflected in the SEM images, consistent with TME images, which showed that I-MgCuBTC exhibits rougher surface morphologies and more irregular morphologies compared to the pristine sample. In the case of P-MgCuBTC (Figure 7c), the morphology appears significantly degraded appearance in SEM, which is consistent with the broadening and shifting of XRD peaks, reduced thermal stability from TGA, altered pore size distribution, and reduced SSA and total pore volume.

In all Ni-modified samples (Figure 7d–h), the particle surfaces appear rougher compared to pristine CuBTC, which is likely due to the partial substitution of Cu^2+^ by Ni^2+^ ions. However, at 40 min of modification (Figure 7h), the sample exhibits signs of particle fragmentation, such as irregular surface texture and fragmentation. This observation correlates well with the broadening and shifting of XRD peaks, reduced porosity from N_2_ sorption data, and lower weight loss indicated by TGA, all of which suggest partial collapse or distortion of the CuBTC framework.^41^

The different influence between Mg^2+^ and Ni^2+^ on CuBTC modification is attributed to their differing chemical characteristics. Ni^2+^, like Cu^2+^, is a transition metal, but it also exhibits multiple spin states and diverse coordination chemistry.^53,54^ These characteristics may enable Ni^2+^ to substitute Cu^2+^ within the framework. In contrast, Mg^2+^ is not a transition metal and has a significantly different electronic configuration compared to Cu^2+^. These differences lead to greater structural incompatibility, particularly in the case of Mg^2+^ modification, resulting in more pronounced disruption of the CuBTC framework in both *in situ* and postsynthetic processes.

## 4 Hydrogen Storage Analysis

To understand the hydrogen storage performance of both pristine and modified CuBTC samples, the hydrogen sorption isotherm measurements were conducted at 77 K using the IGA instrument, as presented in Figure 8a. Among all of the samples, the P-NiCuBTC-30, which contains the highest Ni^2+^ concentration of 1.5 wt %, exhibited the highest hydrogen storage capacity, reaching 4.2 wt % at 20 bar and 77 K. This value represents a 31% improvement compared to the pristine CuBTC, which demonstrated a capacity of 3.2 wt %. This enhancement in hydrogen storage capacity following Ni^2+^ postmodification can be attributed to two key factors. First, the partial substitution of Cu^2+^ by Ni^2+^ within the CuBTC structure, which introduces additional active metal sites within the porous framework for hydrogen adsorption, as supported by the increased hydrogen adsorption isosteric enthalpy compared to pristine CuBTC.^41,55–59^ Second, the introduction of Ni^2+^ can increase the content of Cu^+^, which results from defects as illustrated in the XPS results (see Figure 10d). This is also aligned with its TGA results under N_2_, which revealed defects, manifested as Cu^+^ content, in the Ni-modified samples. Cu­(I)-based MOFs are known to exhibit stronger interaction with hydrogen, therefore improving hydrogen uptake.^60^ The synergistic effects arising from Ni^2+^ substitution of Cu^2+^ within the CuBTC framework—leading to the introduction of additional active metal sites and the additional Cu^+^ species—are key factors contributing to the enhanced performance observed in P-NiCuBTC-30.

**Figure 8:**
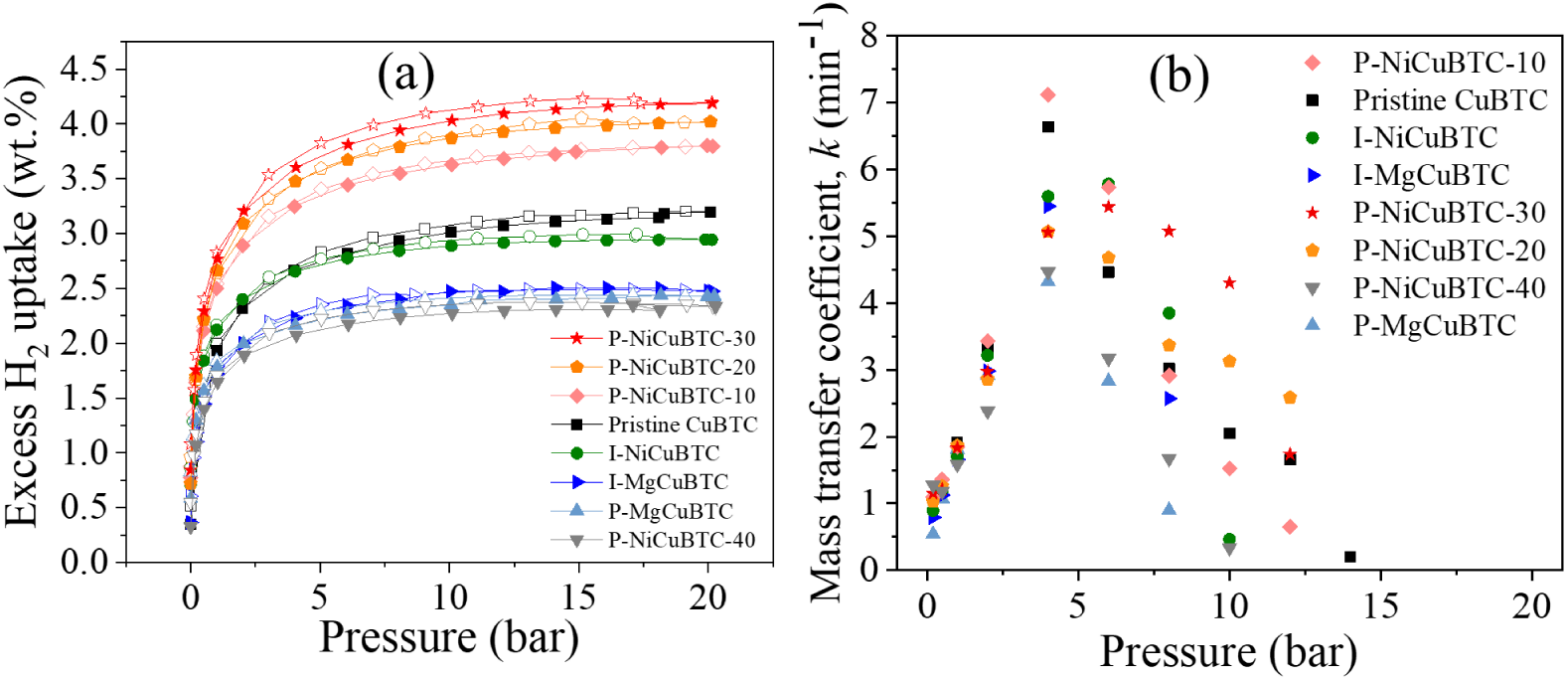
(a) H_2_ adsorption (filled data markers) and desorption (hollow data markers) isotherm at 77 K up to 20 bar; (b) H_2_ adsorption kinetic rate *k* of pristine and modified CuBTC samples (the *k* value approaches zero at dynamic equilibrium, so it is not plotted at that points in Figure 8b).

The hydrogen uptake capacities of both P-MgCuBTC and I-MgCuBTC are significantly lower than that of pristine CuBTC. This reduction is likely attributed to the negative impact of Mg^2+^ addition on the formation of the CuBTC framework, as evidenced by TGA and N_2_ sorption analyses. Notably, P-MgCuBTC exhibits even lower hydrogen uptake than I-MgCuBTC, which can be attributed to structural degradation. This is supported by the broader and shifted XRD peaks, reduced SSA and total pore volume, reduced thermal stability, as well as the degraded particle morphology observed in SEM images (Figure 7c).

The I-NiCuBTC also contains Ni^2+^ species, as suggested by SEM-EDS results and increased weight loss observed in TGA analysis. However, N_2_ sorption analyses revealed that the *in situ* addition of Ni^2+^ negatively affected porosity. As a result of these competing effects, I-NiCuBTC showed slightly lower hydrogen storage capacity than pristine CuBTC but higher than all Mg-modified counterparts.

Post-Ni-modified samples exhibited higher hydrogen uptake than pristine CuBTC, likely due to introduced defects and Ni^2+^ content, supported by SEM-EDS, TEM-EDS and TGA analysis. In contrast, P-NiCuBTC-40 shows clear signs of structural degradation, leading to a substantial reduction in hydrogen uptake. In addition, excluding the degraded P-NiCuBTC-40 sample, the remaining post-Ni-modified samples exhibited higher hydrogen storage capacities than the *in situ*-Ni-modified samples. This enhancement can be attributed to greater Ni^2+^ content in the postmodified samples, as evidenced by SEM-EDS and TGA analysis. Furthermore, it is observed that the Ni^2+^ content in NiCuBTC shows a positive correlation with the hydrogen storage capacity. This is attributed to the Ni^2+^ substitution, which creates additional metal-active sites and Cu^+^ defects that enhance hydrogen adsorption.

As the hydrogen diffusion rate is potentially the rate-limiting step in the overall adsorption process, it is often used to represent the hydrogen adsorption kinetics.^33^ To quantitatively assess this, the diffusion rate constants of the mass transfer coefficients (denoted as “*k*”) were determined using the Linear Driving Force (LDF) model,^61,62^ as described in eq 1. Higher values of *k* indicate a faster hydrogen diffusion rate within the porous structure, which corresponds to a more rapid hydrogen adsorption process.

1 $$m=m_{0}+\Delta m\left[1-\exp\left\{-\left(t-t_{0}\right)/k\right\}\right]$$

where *m* denotes the amount of hydrogen adsorbed (mg), *t* is the time (min), *m*_0_ represents the initial hydrogen uptake (mg) at *t* = *t*_0_, Δ*m* is the change in hydrogen uptake between the initial and equilibrium states, and k is the mass transfer coefficient (min^–1^).

For each pressure dosing step in the hydrogen adsorption isotherm measurements, the LDF model was applied to calculate the corresponding *k* values. Figure 8b presents the variation of *k* as a function of pressure for the pristine CuBTC and all modified samples. For all eight samples, the *k* values are generally comparable, with an increase observed up to approximately 4 bar. This trend is due to the high availability of accessible pores and adsorption sites that facilitate rapid hydrogen uptake at low pressure.^33^

Between 4 and 10 bar, the *k* values gradually decreased, which may be attributed to the progressive saturation of the adsorption sites and pore volume by hydrogen molecules, leading the system toward dynamic equilibrium. Within this pressure range, the influence of the structural modification became evident. As observed, the Mg-modified samples exhibited lower hydrogen adsorption rates compared to pristine CuBTC, consistent with the previously discussed negative effect of Mg^2+^ introduction on the framework and structural integrity. In contrast, all Ni-modified samples—except P-NiCuBTC-40—showed higher hydrogen adsorption rates than pristine CuBTC. This enhancement may be attributed to the partial substitution of Cu^2+^ by Ni^2+^, which introduces additional highly active sorption sites and defect of Cu^+^. P-NiCuBTC-40, however, showed a significantly lower hydrogen adsorption rate, consistent with the structural degradation observed in earlier analyses. At pressures above 10 bar, all samples approached dynamic equilibrium, as indicated by *k* values approaching zero.

Since the P-NiCuBTC-30 sample exhibited the highest hydrogen storage capacity, a high-pressure isotherm measurement up to 150 bar was performed on this sample using the HTP-1 instrument. The hydrogen uptake results obtained from HTP-1 were consistent with those measured by the IGA system (Figure 9a), confirming the reliability and reproducibility of the experimental data.

**Figure 9:**
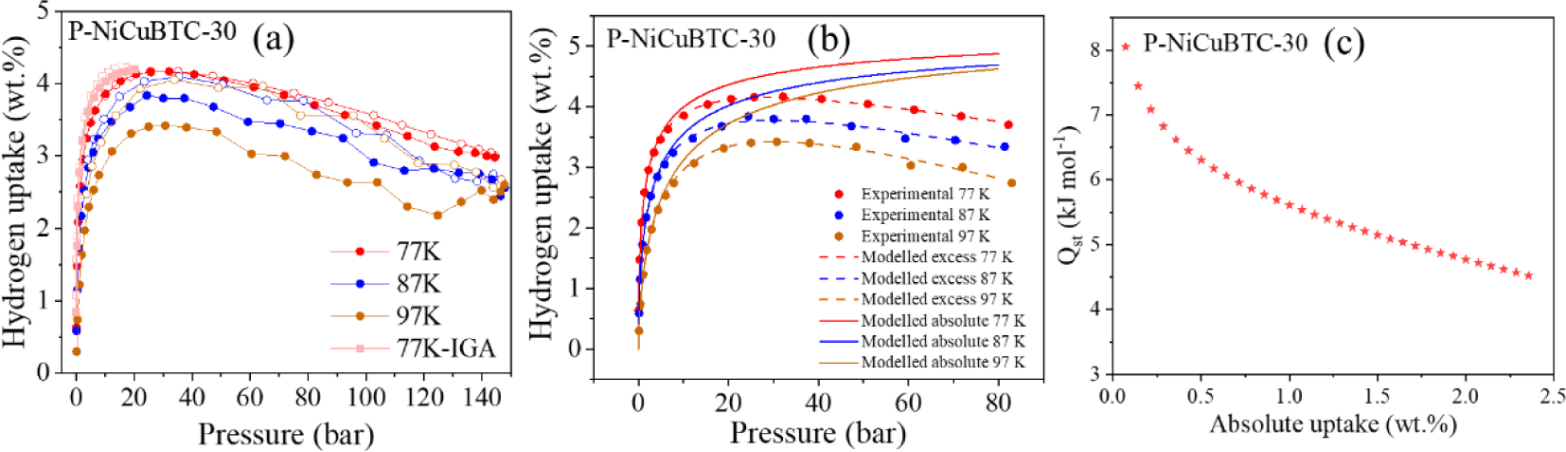
(a) H_2_ adsorption (filled data markers) and desorption (hollow data markers) isotherm at 77 K up to 150 bar; (b) experimental and modeled hydrogen uptake results; (c) Q_st_ results of P-NiCuBTC-30.

Excess uptake is the experimentally measured amount of hydrogen adsorbed, defined as the quantity of gas in the sample in excess of the amount that would be present in the same volume if it contained only bulk gas at the same temperature and pressure. Helium, assumed to be nonadsorbing under measurement conditions, is commonly used to determine the sample’s void volume. Absolute uptake represents the total quantity of hydrogen adsorbed, obtained by adding the adsorbed-phase contribution to the excess uptake, assuming a uniform adsorbed-phase density.^36^ From the experimentally measured excess uptake, the absolute uptake (*n*_*abs*_) of P-NiCuBTC-30 was calculated using the Sips equation,^63^ which combines features of the Freundlich and Langmuir adsorption models,^64^ as shown in Figure 9b and expressed in eq 2.

2 $$n_{abs}=N\times bP^{K}/\left(1+bP^{K}\right)$$

where *N* denotes the maximum adsorption capacity of P-NiCuBTC-30 at a specific temperature, *b* corresponds to the equilibrium constant associated with the Langmuir adsorption coefficient, and *K* represents the heterogeneity factor linked to the Freundlich isotherm. The Sips equation reduces to the Freundlich equation when either the pressure (*P*) or the constant *b* approaches zero. Conversely, when *K* equals 1, the equation simplifies to the classical Langmuir isotherm.^65^

Then the isosteric heat of adsorption of (*Q*_st_, kJ mol^–1^) at a given absolute hydrogen uptake was calculated using the Clausius–Clapeyron equation, as expressed in eq 3:

3 $$\ln P=\left(Q_{st}/R\right)\times \left(1/T\right)$$

where *P* is the equilibrium pressure, *T* is the absolute temperature at a given absolute hydrogen uptake, and *R* is the molar gas constant (8.314 J mol^–1^ K^–1^). This approach allows for the estimation of the adsorption enthalpy based on pressure–temperature relationships at constant adsorbed amounts.

The results of the isosteric heat of adsorption *Q*_st_ for P-NiCuBTC-30 were plotted as a function of absolute hydrogen uptake (up to 2.4 wt.%), as shown in Figure 9c. The *Q*_st_ values ranged from 8.05 to 4.5 kJ mol^–1^, which are notably higher than the typical *Q*_st_ range reported for MOFs (6.6–4.5 kJ mol^–1^)^31^ and also exceed the reported value for pristine CuBTC (5.9 kJ mol^–1^) at low coverage.^66^ These elevated Q_st_ suggest that the partial substitution of Cu^2+^ by Ni^2+^ enhances the interaction strength between hydrogen molecules and the MOFs, due to the increased number of exposed metal sites. In addition, the presence of more accessible exposed metal binding sites for hydrogen molecules contributed to the higher hydrogen uptake observed in the modified sample compared to pristine CuBTC.^67^

## 5 Modification Mechanism

XPS was applied to analyze the elemental composition and chemical speciation of pristine CuBTC and P-NiCuBTC-30.^68^ Figure 10a displays the XPS survey spectra of both samples, where three main peaks appear at 931, 532, and 284 eV, corresponding to Cu 2p, O 1s, and C 1s, respectively. In P-NiCuBTC-30, the spectra shape, multiplet splitting structure and auger, confirms the successful incorporation of Ni^2+^ through the postmodification process. The spectra was fitted using a NiO model derived from a standard Ni sample.

**Figure 10:**
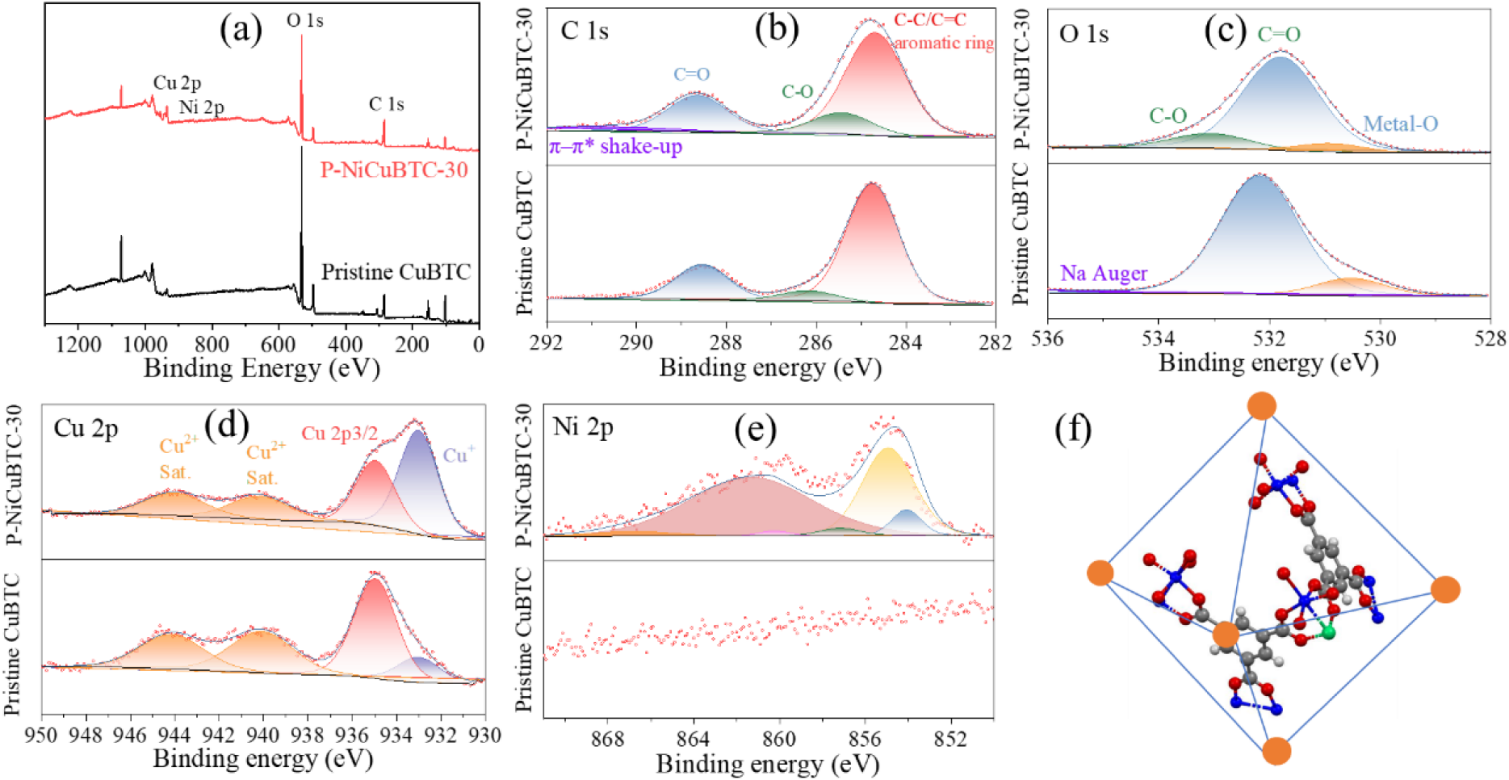
(a–e) XPS spectra of pristine CuBTC and nickel postmodified for 30 min, P-NiCuBTC-30, (f) nickel postmodified for 30 min, P-NiCuBTC-30 structure (carbon atom: gray ball; hydrogen atom: white ball, oxygen atom: red ball; copper atom: blue ball; nickel atom: green ball).

The high-resolution C 1s spectrum in Figure 10b reveals several distinct peaks. The main peak at 284.8 eV is attributed to the carbon–carbon bond in the aromatic rings of the benzene-1,3,5-tricarboxylic acid ligand. The peak at approximately 286.2 eV is attributed to C–O bonds, while the peak at 288.5 eV is assigned to C=O groups. In the case of P-NiCuBTC-30, there is a π–π* transition associated with ligand conjugation.^69^

In the O 1s spectrum (Figure 10c), the peak at 531.8 eV is assigned to C=O groups, and the peak at 533.1 eV corresponds to C–O bonds. A lower binding energy peak at approximately 530.0 eV is associated with metal–oxygen bonds, including both Cu–O and Ni–O; however, these are difficult to distinguish from each other due to their overlapping energies. Additionally, a small signal near 536 eV in the pristine CuBTC sample is attributed to Na Auger signals. In the pristine CuBTC sample, the intensity ratio between the ∼530 eV and ∼532 eV peaks are approximately 1:8, whereas after 30 min of postmodification (P-NiCuBTC-30), this ratio shifts to about 1:12, indicating the incorporation of oxygen into the organic linker. Additionally, in P-NiCuBTC-30, the partial substitution of Cu^2+^ by Ni^2+^ alters the local oxygen coordination environment, leading to a shift in the binding energy of the C=O groups and the formation of C–O related defects. This structural modification may account for the increased proportion of Cu^+^ defects observed in this sample.

Figure 10d shows the high-resolution Cu 2p XPS spectra of both samples within the binding energy range of 950–930 eV. Cu 2p has two main peaks, one for Cu^+^ (933)^70^ and another for Cu^2+^ (934.8),^71^ where the satellite structure confirms the presence of a nonmetallic species. Auger spectra further suggest the mixing of oxidation states. Notably, after Ni^2+^ substitution, the Cu^+^ peak intensity in P-NiCuBTC-30 is greater than the pristine CuBTC. Specifically, the Cu^+^/Cu^2+^ ratio of P-NiCuBTC-30 increases to 62%, compared to 20% in pristine CuBTC, representing a 42% increase, as reported in Table S3. This increase in Cu^+^ concentration correlates with the enhanced hydrogen storage capacity. The presence of Cu^+^ is commonly associated with structural defects,^66^ which in this case are likely introduced by both the Ni^2+^ substitution and the ball milling modification process. This observation is consistent with the TGA-DTG results.

Figure 10e displays the high resolution of Ni 2p XPS spectra of the two samples in the range of 891–850 eV. As expected, pristine CuBTC does not exhibit any Ni-related peaks. In contrast, for P-NiCuBTC-30, the Ni 2p spectra confirm the presence of NiO, with the multiplet splitting structure fitting well within the expected compound. Auger for Ni 2p further confirms the NiO assignment. Both XPS and Auger feature similar spectral structures to what is reported by Biesinger et al.^72^ This confirms that Ni^2+^ successfully replaced Cu^2+^ and formed coordination bonds with O atoms within the CuBTC framework. This proposed structural model is illustrated schematically in Figure 10f.

In addition, as seen in Table S2, the Ni^2+^ concentration obtained from the XPS analysis for P-NiCuBTC-30 is 1.1 wt.%, which is close to the SEM-EDS value of 1.5 wt.%, confirming the consistency of the two characterization methods and reflecting the actual Ni^2+^ content in the material.

## 6 Conclusion

This study provides the first demonstration of the mechanochemical modification of CuBTC (also known as HKUST-1), a metal–organic framework (MOF), using Mg^2+^ and Ni^2+^ through a combination of *in situ* and postmodification ball milling methods. FTIR analysis confirms that the characteristic coordination features of the CuBTC framework remain intact, indicating that the introduced amount of metal does not disrupt the primary coordination environment. The XRD results show that the original crystal structure is largely preserved after metal introduction, except in nickel-modified CuBTC (P-NiCuBTC-40) and magnesium-modified CuBTC (P-MgCuBTC), which exhibit peak broadening and shifting due to the degradation.

The TGA and DTG thermal analyses reveal that Mg^2+^ was not incorporated into the CuBTC framework, whereas Ni^2+^ partially replaced Cu^2+^ within the structure. This conclusion is proved by both SEM-EDS and TEM-EDS results. The post-Ni-modification method generally introduces a greater substitution of Cu^2+^ by Ni^2+^ and structural defects compared to the *in situ*-Ni-modification method, as evident by SEM-EDS, TEM-EDS and TGA analysis. Among the Ni-modified samples, P-NiCuBTC-30 demonstrates the highest Ni^2+^ content and Cu^+^ defects, and enhanced thermal stability, indicating an optimal balance between metal substitution and structural preservation.

N_2_ sorption analysis at 77 K shows that all metal-modified CuBTC samples retain the characteristic dual-step isotherm and two main pore sizes, but exhibit reduced N_2_ adsorption capacity, surface area, and pore volume compared to pristine CuBTC. In the Mg-modified samples, the *in situ* magnesium-modified CuBTC (I-MgCuBTC) exhibits reduced porosity due to interference with framework formation, while the magnesium postmodified CuBTC (P-MgCuBTC) suffers from degradation compared to pristine CuBTC. Similarly, Ni^2+^ modified CuBTC displays progressively reduced porosity originating from Cu^2+^ substitution by Ni^2+^ and structural defects of Cu^+^. P-NiCuBTC-40 exhibits significant structural degradation, resulting in the lowest porosity among Ni-modified samples.

SEM and TEM images show that pristine CuBTC has a smooth, well-defined octahedral morphology, while the introduction of Mg^2+^ and Ni^2+^ causes rougher, less regular particles. P-MgCuBTC and P-NiCuBTC-40 exhibit the most severe structural degradation, consistent with left shift and broad XRD peaks, and reduced porosity.

Among all the samples evaluated, the P-NiCuBTC-30, the highest Ni^2+^ concentration of 1.5 wt %, exhibited the highest hydrogen storage capacity of 4.2 wt % at 20 bar and 77 K. This represents a 31% enhancement compared to the pristine CuBTC under identical conditions. This enhancement is attributed to the creation of additional adsorption sites and the increase of Cu^+^ content induced by the Ni^2+^ substitution. Overall, the post-Ni-modified samples exhibited higher hydrogen uptake than the *in situ*-Ni-modified samples, which results from their higher density of Ni^2+^ content and structural defects of Cu^+^.

In summary, a combination of detailed structural and adsorption–desorption characterization has been employed to examine and understand the impact of introducing divalent metal ions of Ni^2+^ and Mg^2+^ through *in situ* and postmodification methods. We observe that in the post-Ni-modified sample, the substitution of Cu^2+^ by Ni^2+^ increases the number of active metal adsorption sites and promotes the formation of Cu^+^ defect sites, both of which contribute to the significantly enhanced hydrogen storage capacity compared to pristine CuBTC. These findings indicate that post-Ni modification via a solvent-free mechanochemical route is an effective new strategy for improving hydrogen storage performance. As a result, this work highlights the promise of dual-metal frameworks in overcoming current limitations in physisorptive hydrogen storage and brings us closer to practical, scalable solid-state hydrogen storage solutions.

## Supplementary Material



## Notes

Additional characterization details, including SEM-EDS, TEM-EDS, XPS, and an example of the XPS fitting procedure (PDF)

## Abbreviations

MOFs: metal–organic frameworks
CuBTC: copper benzene-1,3,5-tricarboxylate
HBTC: benzene-1,3,5-tricarboxylic acid
DMSO: dimethyl sulfoxide
Mg­(NO_3_)_2_·6H_2_O: magnesium nitrate hexahydrate
Ni­(NO_3_)_2_·6H_2_O: nickel nitrate hexahydrate
H_2_: hydrogen
CO_2_: carbon dioxide
N_2_: nitrogen
He: helium
Cr: chromium
SSA: specific surface area
GCMC: grand canonical Monte Carlo
Sc: scandium
Ni: nickel
Cr: chromium
Ti: titanium
Mn: manganese
V: vanadium
SBU: secondary building unit
XRD: X-ray diffraction
FTIR: Fourier-transform infrared spectroscopy
SEM: scanning electron microscopy
TGA: thermogravimetric analysis
DTG: derivative thermogravimetry
XPS: X-ray photoelectron spectroscopy
BET: Brunauer–Emmett–Teller
NLDFT: nonlocal density functional theory
FCC: face-centered cubic
LDF: linear driving force
