# Supplementary material for: Mechanochemical Dual-Metal Modification of CuBTC Metal–Organic Frameworks for Enhanced Hydrogen Storage
Source: ACS Appl Mater Interfaces. 2025 Dec 8;17(50):68703–16. doi: 10.1021/acsami.5c18519 (PMC12723631; doi:10.1021/acsami.5c18519)
Supplement: Supplementary file 1 [file am5c18519_si_001.pdf]

Supporting Information

Mechanochemical dual-metal modification of CuBTC metal-organic framework for enhanced hydrogen storage

Qian Yu[a], Charles D. Brewster[b], Rajan Jagpal[b], Arthur Graf[c, d], Xiayi Hu[e], Timothy J Mays[b], Mi Tian[b*]

[a] Department of Engineering, Faculty of Environment, Science and Economy, University of Exeter, Streatham Campus, Exeter, EX4 4QJ, UK.

[b] Department of Chemical Engineering, Faculty of Engineering & Design, University of Bath, Claverton Down, Bath, BA2 7AY, UK.

[c] HarwellXPS, Research Complex at Harwell, Harwell Campus, Didcot, OX11 0FA, UK

[d] Department of Chemistry, Cardiff University, Cardiff, CF10 3AT, UK

[e] Chemical Engineering and Technology, Xiangtan University, Xiangtan, Hunan, 411105, China

*Email: mt747@bath.ac.uk Telephone: +4407916578189

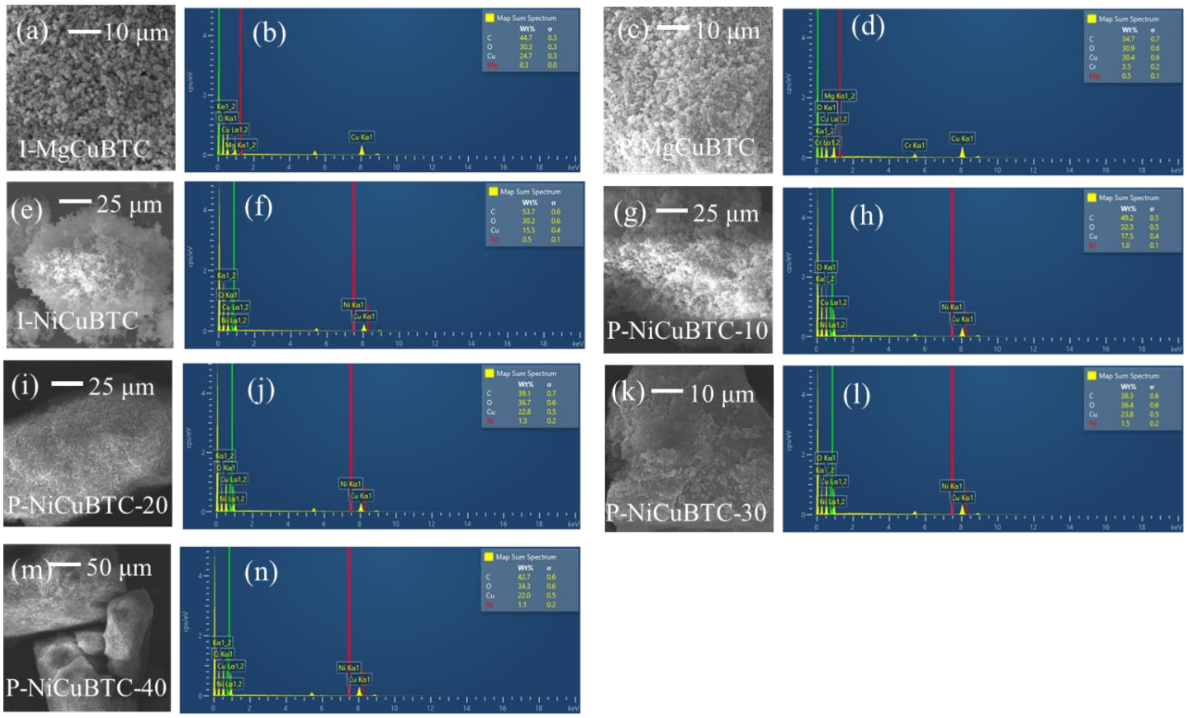

Figure S1. SEM images and corresponding EDS elemental analysis of (a, b) in situ Mg-modified I-MgCuBTC, (c, d) post-modified P-MgCuBTC, (e, f) in situ Ni-modified I-NiCuBTC, and post-Ni-modified samples (g, h) P-NiCuBTC-10, (i, j) P-NiCuBTC-20, (k, l) P-NiCuBTC-30, and (m, n) P-NiCuBTC-40.

Table S1. TEM-EDS elemental analysis of P-NiCuBTC-30

Element	Weight %	Atomic %
Ni	0.14	0.16
Cu	99.86	99.84

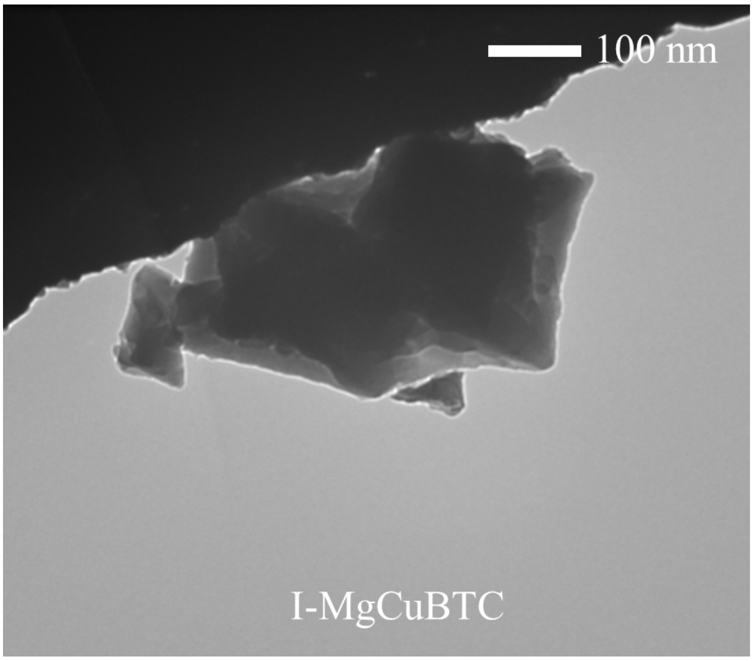

Figure S2. TEM image of I-MgCuBTC adhering to the copper grid edge

Table S2. XPS elemental analysis of P-NiCuBTC-30

Name	Position	Full width at half maximum	Raw area	Concentration (wt.%)
C 1s	284.8	1.71	17365.25	61.03
O 1s	531.08	1.87	27676.12	31.30
Cu 2p	933.2	3.45	54187.08	6.54
Ni 2p	885.20	0.20	8089.10	1.12

Table S3. Cu^{+}/Cu^{2+} ratio comparison between pristine CuBTC and P-NiCuBTC-30.

Sample name	Raw area of Cu^{+}	Raw area of Cu^{2+}	Cu^{+}/Cu^{2+} ratio
Pristine CuBTC	6324.8	25140.9	20 %
P-NiCuBTC-30	24072	15031.3	62 %

Figure S3 illustrates the XPS fitting procedure using the C 1s spectrum of pristine CuBTC as an example. The fitting process consists of three main steps: (i) defining the fitting region (Figure S3a), (ii) assigning three component peaks corresponding to the carbon environments (Figure S3b), and (iii) calibrating the binding energies using the C-C peak at 284.8 eV as the reference (Figure S3c).

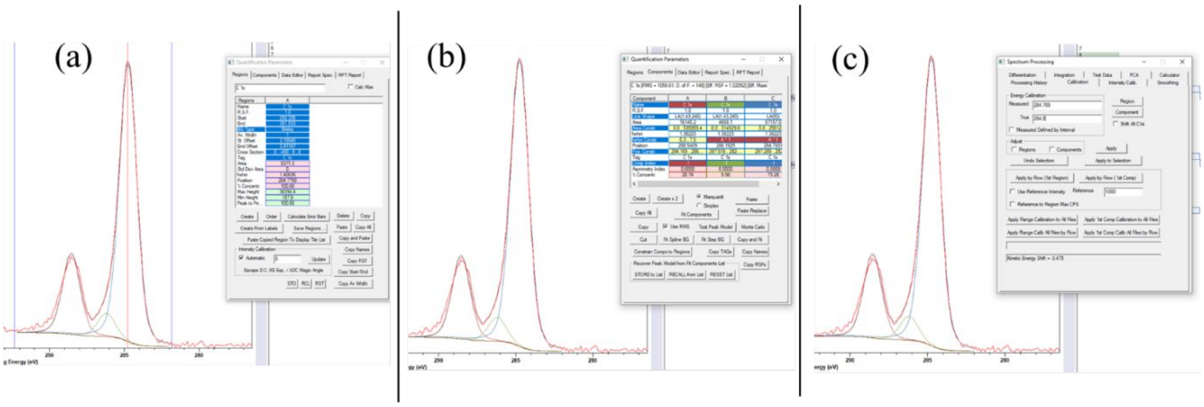

Figure S3. XPS fitting procedures using the C 1s spectrum of pristine CuBTC as an example.
